# Activation of NF‐κB Signaling by Optogenetic Clustering of IKKα and β

**DOI:** 10.1002/adbi.202400384

**Published:** 2025-07-29

**Authors:** Alexandra Anna Maria Fischer, Markus Michael Kramer, Miguel Baños, Merlin Moritz Grimm, Manfred Fliegauf, Bodo Grimbacher, Gerald Radziwill, Sven Rahmann, Wilfried Weber

**Affiliations:** ^1^ Signalling Research Centers BIOSS and CIBSS University of Freiburg Schänzlestr. 18 79104 Freiburg Germany; ^2^ Faculty of Biology University of Freiburg Schänzlestr. 1 79104 Freiburg Germany; ^3^ Spemann Graduate School of Biology and Medicine (SGBM) University of Freiburg Albertstr. 21a 79104 Freiburg Germany; ^4^ INM – Leibniz Institute for New Materials Campus D2 2 66123 Saarbrücken Germany; ^5^ Department of Materials Science and Engineering Saarland University Campus D2 2 66123 Saarbrücken Germany; ^6^ Institute for Immunodeficiency Center for Chronic Immunodeficiency (CCI) Medical Center, Faculty of Medicine Albert‐Ludwigs‐University of Freiburg Breisacherstr. 115 79106 Freiburg Germany; ^7^ DZIF – German Center for Infection Research Satellite Center Freiburg Breisacherstr. 115 79106 Freiburg Germany; ^8^ RESIST – Cluster of Excellence 2155 to Hanover Medical School Satellite Center Freiburg Breisacherstr. 115 79 106 Freiburg Germany; ^9^ CIBSS – Centre for Integrative Biological Signalling Studies University of Freiburg Schänzlestr. 18 79104 Freiburg Germany; ^10^ Algorithmic Bioinformatics Center for Bioinformatics Saar and Saarland University Saarland Informatics Campus E2.1 66123 Saarbrücken Germany

**Keywords:** NF‐κB signaling, oligomerization, optogenetics, phase separation, synthetic biology

## Abstract

Molecular optogenetics allows the control of molecular signaling pathways in response to light. This enables the analysis of the kinetics of signal activation and propagation in a spatially and temporally resolved manner. A key strategy for such control is the light‐inducible clustering of signaling molecules, which leads to their activation and subsequent downstream signaling. In this work, an optogenetic approach is developed for inducing graded clustering of different proteins that are fused to eGFP, a widely used protein tag. To this aim, an eGFP‐specific nanobody is fused to Cryptochrome 2 variants engineered for different orders of cluster formation. This is exemplified by clustering eGFP‐IKKα and eGFP‐IKKβ, thereby achieving potent and reversible activation of NF‐κB signaling. It is demonstrated that this approach can activate downstream signaling via the endogenous NF‐κB pathway and is thereby capable of activating both an NF‐κB‐responsive reporter construct as well as endogenous NF‐κB‐responsive target genes as analyzed by RNA sequencing. The generic design of this system is likely transferable to other signaling pathways to analyze the kinetics of signal activation and propagation.

## Graphical abstract

This study presents an optogenetic approach for graded clustering of eGFP‐fused proteins using an eGFP‐specific nanobody and engineered Cryptochrome 2 variants. The method enables potent, reversible activation of NF‐κB signaling via endogenous pathways, as confirmed by RNA sequencing. This versatile system provides a spatially and temporally controlled tool for studying signal activation kinetics in diverse molecular pathways.

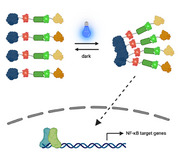

## 1 Introduction

Protein oligomerization is an essential mechanism for the control of cellular signaling processes and a large percentage of cellular proteins oligomerize in order to fulfill their function^[^ ^1^, ^2^, ^3^ ^]^ Their correct assembly, in terms of location, time, order of oligomerization, and binding partners, decides critically over the signaling output.^[^ ^4^ ^]^ Investigation of these processes requires sophisticated research tools capable of tuning oligomerization. A versatile method to achieve this is molecular optogenetics. This method uses bacteria‐ or plant‐derived photoreceptors, genetically fused to cellular effector proteins, and thereby enables control over their function using light of a specific wavelength as a non‐invasive, reversible, local, and dose‐dependent stimulus. The optogenetics toolbox provides a large variety of photoswitches that allow the assembly of a protein of interest into homo‐or heterodimers,^[^ ^5^, ^6^, ^7^, ^8^, ^9^ ^]^ homo‐ or heterooligomers,^[^ ^10^, ^11^, ^12^ ^]^ or phase‐separated biomolecular condensates.^[^ ^13^, ^14^, ^15^ ^]^ The most established self‐oligomerizing photoreceptor is Cryptochrome 2 (Cry2).^[^ ^10^ ^]^ Its variants, Cry2_olig_^[16]^ or Cry2_clust,_^[17]^ can form higher‐order homo‐oligomers, and furthermore, they can all form heterooligomers with their binding partner CIBN^[^ ^5^ ^]^ with highly resolved spatio‐temporal precision.^[^ ^15^, ^17^, ^18^ ^]^ Combining Cry2 with an intrinsically disordered region (IDR) such as the N‐terminal domain of Fused in Sarcoma (FUS_N_) leads to the formation of phase‐separated liquid‐like biomolecular condensates, called optoDroplets, after blue light illumination. The exchange of Cry2 with the Cry2_olig_ variant leads to the formation of larger, solid‐like gels.^[^ ^15^ ^]^

Cry2, fused to different signaling molecules, has been used to dynamically control signaling pathways,^[^ ^19^, ^20^ ^]^ such as the mitogen‐activated protein kinase (MAPK)^[^ ^21^, ^22^, ^23^, ^24^ ^]^ pathway, Wnt/β‐catenin signaling,^[^ ^10^, ^25^ ^]^ receptor tyrosine kinase^[^ ^26^, ^27^, ^28^ ^]^ activated signaling pathways, or recently NF‐κB signaling by clustering MyD88 and TRAF6.^[^ ^18^, ^29^ ^]^

NF‐κB signaling is regulated by higher‐order oligomerization events at several levels of the pathway.^[^ ^30^ ^]^ It is activated by various factors, among them TNF‐α which binds to the tumor necrosis factor receptor (TNFR). Stimulation of the TNFR with TNF‐α, leads to receptor trimerization and the recruitment of a higher‐order complex consisting, among others, of adaptor proteins serine/threonine‐kinases and E2/E3 ubiquitin ligases that catalyze the formation of polyubiquitin chains. The TAK1/TAB2/TAB1 complex is recruited to the polyubiquitin chains, as well as the IκB kinase (IKK) complex. The IKK complex represents the central signal integration module of the NF‐κB signaling pathway and consists of the regulatory subunit NEMO, which acts as a scaffold to assemble the two serine‐threonine kinases IKKα and IKKβ. Recently, it has been discovered that the binding of the polyubiquitin chains (polyUb) to NEMO leads to the formation of phase‐separated biomolecular condensates that are crucial for efficient downstream signaling activation. They harbor TAK1 as well as IKKα and β, thereby likely facilitating efficient IKK phosphorylation and activation by TAK1.^[^ ^31^, ^32^ ^]^ Several studies describe IKK activation by trans‐autophosphorylation of IKKα and β. Subsequently, IKKβ phosphorylates IκBα, which is then ubiquitinated and degraded. This releases the NF‐kB transcription factor dimer (e.g., RelA and p50), which translocates to the nucleus and activates gene expression.^[^ ^33^, ^34^, ^35^, ^36^ ^]^

Usually, optogenetic tools require the fusion of a photoreceptor to a protein of interest, which requires recloning for every new target protein. In this study, we engineered a toolbox of constructs to cluster proteins fused to eGFP, which has widely been used to tag arbitrary proteins. To this aim, we used the blue‐light inducible oligomerization of the photoreceptor Cry2 and fused it to an eGFP‐specific nanobody (NbGFP)^[^ ^37^ ^]^ for recruitment of eGFP‐tagged proteins. We combined NbGFP with Cry2, Cry2_olig,_^[16,^ ^38^, ^39^ ^]^ or the phase separation‐inducing optoDroplet configuration (Cry2/Cry2_olig_‐FUS_N_)^[^ ^15^ ^]^ to achieve graded clustering. We applied the constructs to cluster eGFP‐tagged IKKα and β, as their role as central signaling integrators may allow control of the NF‐κB signaling pathway. Using these constructs, we uncoupled pathway activation from upstream signaling events and engineered a blue‐light inducible, optogenetic activator of the NF‐κB pathway.

## 2 Results

### 2.1 Design of an Optogenetic Toolbox to Gradually Cluster eGFP‐Tagged Proteins

**Figure 1:**
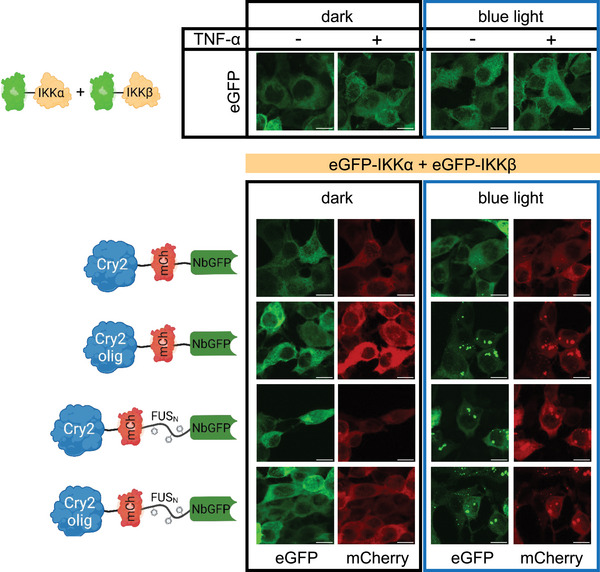
Microscopical characterization of the clustering constructs. HEK‐293T cells were transfected with the indicated constructs. Blue‐light illumination (5 µmol m^−2 ^s^−1^) was started 8 h after transfection, and samples were fixed for microscopy analysis 24 h later. Representative images are shown; scale bar = 10 µm.

We envisioned a toolbox of optogenetic constructs that enables convenient targeting and clustering of proteins of interest to different degrees. To this aim, we fused the photoreceptor Cry2 to mCherry (mCh) for visualization, a nanobody that can specifically bind to the fluorescent protein eGFP (NbGFP), and a nuclear export sequence (NES) to direct the fusion protein to the cytoplasm (Cry2‐mCh‐NES‐NbGFP). The protein of interest was fused to eGFP, which is a widely used fusion partner.^[^ ^38^, ^40^, ^41^ ^]^ Starting from this basic design, we changed Cry2 to Cry2_olig_ to increase clustering (Cry2_olig_‐mCh‐NES‐NbGFP) and in the next step, inspired by the optoDroplet approach,^[^ ^15^ ^]^ incorporated FUS_N_. We hypothesized that this would lead to the formation of phase‐separated biomolecular condensates after blue‐light illumination (Cry2‐mCh‐FUS_N_‐NES‐NbGFP and Cry2_olig_‐mCh‐FUS_N_‐NES‐NbGFP, **Figure** **1**). To exemplify this approach, we selected IKKα and IKKβ as target proteins and tagged them with eGFP. To validate the expression and localization of the system components, we transfected eGFP‐IKKα and eGFP‐IKKβ in HEK‐293T cells together with one of the cluster‐inducing constructs and kept them either in darkness or under blue light illumination. Both eGFP‐IKKα and eGFP‐IKKβ, as well as the clustering constructs, localized to the cytoplasm, and all constructs showed a diffuse signal in darkness. Upon blue light illumination, we observed a low number of small clusters for Cry2‐mCh‐NES‐NbGFP and large cytoplasmic clusters for Cry2_olig_‐mCh‐NES‐NbGFP, Cry2‐mCh‐FUS_N_‐NES‐NbGFP, and Cry2_olig_‐mCh‐FUS_N_‐NES‐NbGFP in both mCherry and eGFP channels, demonstrating the binding and recruitment of IKKα/β into the clusters (Figure 1).

### 2.2 Optogenetic Clustering of IKKα and IKKβ Activates NF‐kB Signaling Independent of Upstream Signals

**Figure 2:**
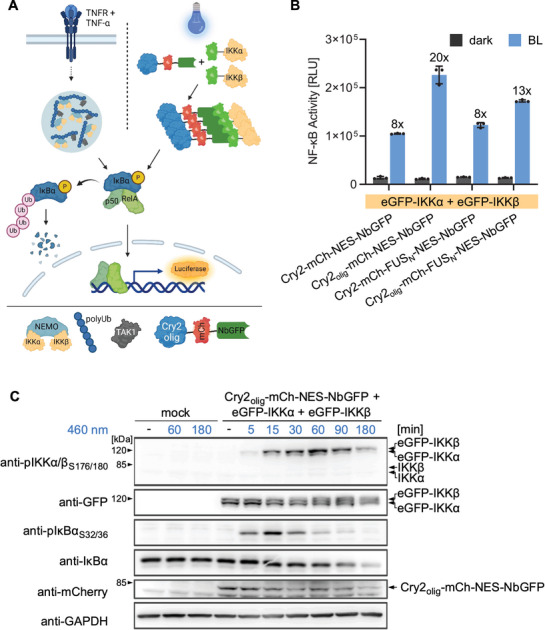
Optogenetic activation of NF‐κB signaling. A) Schematic depiction of endogenous and optogenetic NF‐κB pathway activation. The TNFR is activated upon binding of TNF‐α. After intermediate steps (indicated by the dashed line), binding of NEMO to polyUb chains leads to the formation of biomolecular condensates that also recruit the kinase TAK1 and the IKK complex through binding to the polyUbs or NEMO respectively. The IKK complex is activated and subsequently phosphorylates IκBα, which is then ubiquitinated and degraded. This releases the NF‐κB transcription factor dimer RelA/p50, which translocates to the nucleus and activates gene expression. In the optogenetic approach, we cluster eGFP‐tagged IKKα and IKKβ and thereby activate the pathway independently from upstream processes. B) Blue‐light induced activation of NF‐κB signaling. HEK‐293T cells were transfected with the indicated constructs and an NF‐κB‐responsive firefly luciferase reporter. Blue‐light illumination (5 µmol m^−2 ^s^−1^) began 8 h after transfection and firefly luciferase activity was determined 24 h later. Mean ± SD of a representative assay is shown (*N* = 3). Fold NF‐κB activation, calculated from the respective dark and blue light (BL) samples, is shown above each BL bar. Means ± SD are plotted. C) NF‐κB signal transduction kinetics. HEK‐293T cells were transfected with the indicated constructs, kept in darkness for 24 h, and then illuminated with blue light (5 µmol m^−2 ^s^−1^) for the indicated durations. Immediately afterward they were lysed and analyzed via Western blot.

We hypothesized that optically induced clustering of IKKα and β might be sufficient to activate the kinases independent from upstream activators (**Figure** **2**A). We tested this hypothesis by transfecting HEK‐293T cells with eGFP‐IKKα and eGFP‐IKKβ, together with each construct of the clustering toolbox and an NF‐κB‐responsive firefly luciferase reporter.^[^ ^42^ ^]^ Indeed, blue‐light illumination led to strong activation of reporter expression (Figure 2B), and the activation strength depended on the clustering construct. Cry2_olig_‐mCh‐NES‐NbGFP, which leads to higher‐order oligomerization, resulted in the highest total signal and highest fold induction of NF‐κB activity (Figure 2B). To ensure that these differences indeed derived from different clustering behaviors and not from different expression levels of the clustering constructs, we verified their expression levels by measuring eGFP and mCherry fluorescence via flow cytometry. We found that at least 97% of eGFP‐positive cells were also mCherry‐positive, and no or only minor significant differences in mCherry expression levels between the four different constructs were detected (Figure S1A,B, Supporting Information). Comparing the optogenetically induced reporter signal to the TNF‐α‐induced reporter signal, we obtained only 50–60% of overall reporter expression levels but similar fold inductions. This was due to reduced background reporter expression levels in the presence of the optogenetic constructs likely caused by the binding of NbGFP to eGFP‐IKKα and β (Figure 2B; Figure S2, Supporting Information).

**Figure 3:**
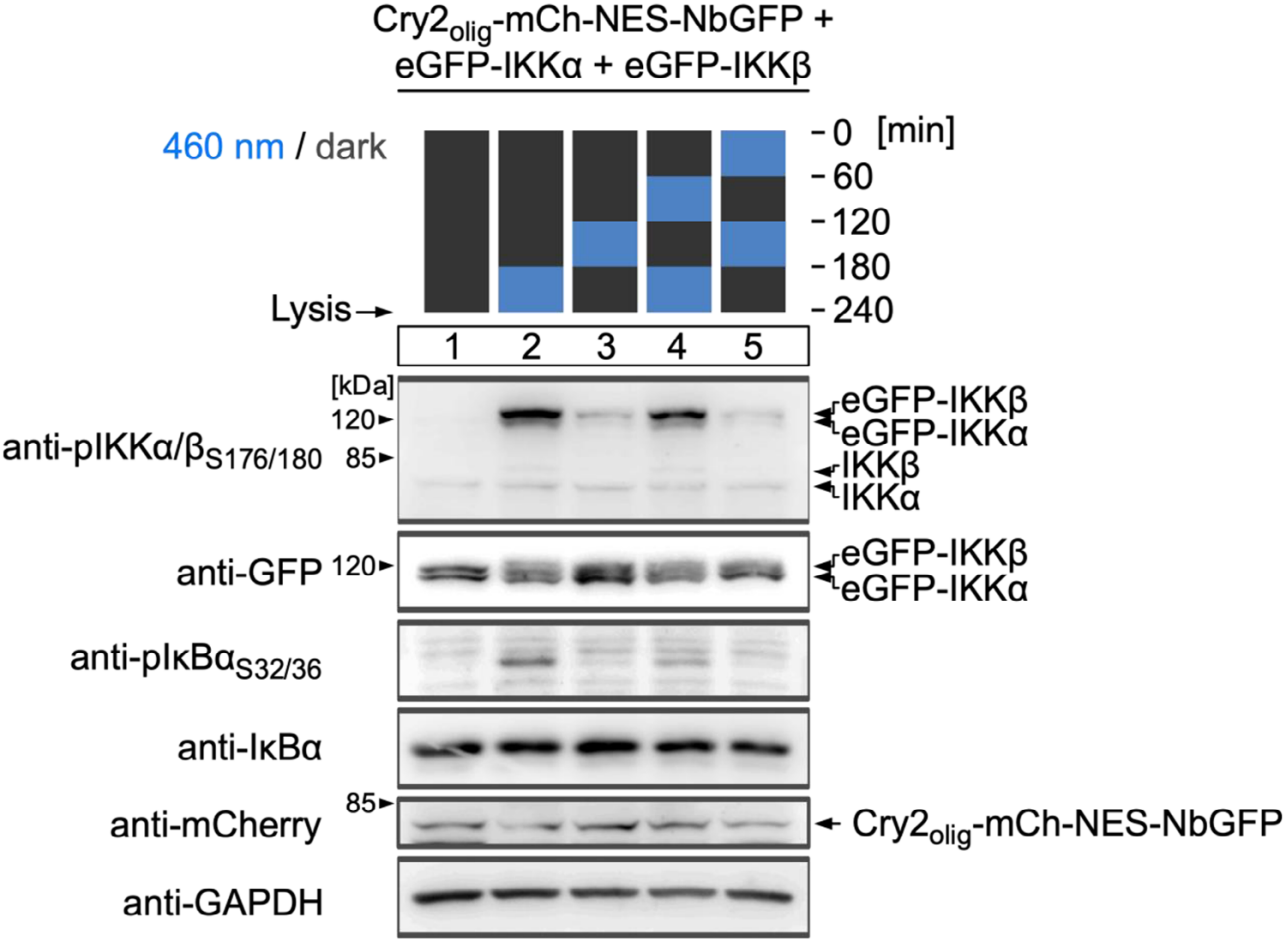
Reversibility of NF‐κB pathway activation. HEK‐293T cells were transfected with the indicated constructs, kept in darkness for 24 h, and then illuminated with blue light (5 µmol m^−2 ^s^−1^) in the indicated temporal pattern. Cells were lysed immediately after illumination and analyzed via Western blot.

Furthermore, we tested if expressing only eGFP‐IKKα or eGFP‐IKKβ is sufficient to activate NF‐κB signaling. Indeed, clustering of eGFP‐IKKα or eGFP‐IKKβ alone was sufficient to activate NF‐κB signaling. This is in line with a previous study by Inohara et al.^[^ ^43^ ^]^ that used a chemically inducible system. However, eGFP‐IKKα led to a much higher signal than eGFP‐IKKβ (Figure S3A, Supporting Information). Analyzing the abundance of the two constructs via flow cytometry and Western blot, we found that eGFP‐IKKα was 1.4‐fold higher expressed than eGFP‐IKKβ, which might contribute to the higher NF‐κB activity of this condition (Figures 2C,3; Figures S3B,C, Supporting Information). Cotransfection of both eGFP‐IKKα and eGFP‐IKKβ led to higher NF‐κB activation than eGFP‐IKKα alone; therefore, and to recapitulate the native system better, we performed the subsequent experiments cotransfecting both.

Clustering of IKKα and β leads to phosphorylation of both molecules at S176/180 and of IκBα at S32/36 and subsequent degradation of IκBα.^[^ ^36^ ^]^ To evaluate whether optogenetic clustering recapitulated these events, we analyzed phosphorylation and degradation via Western blot. We transfected eGFP‐IKKα and eGFP‐IKKβ together with Cry2_olig_‐mCh‐NES‐NbGFP into HEK‐293T cells and induced clustering via blue light illumination for 5, 15, 30, 60, 90, and 180 min. Western blot analysis revealed phosphorylation of eGFP‐IKKα and eGFP‐IKKβ 5 min after blue‐light illumination, reaching a peak at 60 min. Endogenous IκBα phosphorylation was detectable after 5 min, peaking after 15 min. IκBα levels began to decrease after 30 min of illumination and progressively declined over the course of 180 min (Figure 2C). This kinetic is slower than reported in literature, where IκBα degradation within 15 min is described but is likely highly cell line and expression level dependent.^[^ ^44^, ^45^ ^]^

One key advantage of light over chemical stimuli is the opportunity to control temporal kinetics in a fully reversible manner.^[^ ^20^ ^]^ Turning light on and off allows mimicking transient pathway stimulation and investigation of the pathway's dynamics. To test the reversibility of the optogenetic NF‐κB pathway activation, we transfected eGFP‐IKKα and eGFP‐IKKβ together with Cry2_olig_‐mCh‐NES‐NbGFP into HEK‐293T cells. We applied five different illumination patterns of on and off periods (1 h each) for a total duration of 4 h (**Figure** **3**). We observed strong phosphorylation of eGFP‐IKKα, eGFP‐IKKβ, and endogenous IκBα if the cells were lysed directly after a blue‐light illumination step. These phosphorylations were close to background levels when the cells were subjected to a 1 h dark period before lysis, thus confirming the reversibility of the optogenetic phosphorylation of eGFP‐IKKα, eGFP‐IKKβ, and downstream endogenous IκBα.

We concluded from these characterizations that clustering of IKKα and β is sufficient to activate NF‐κB signaling independent from upstream signaling via endogenous IκBα in a rapid and reversible manner, with a similar fold‐induction as by TNFα stimulation.

### 2.3 Activation of Endogenous NF‐κB Target Genes

**Figure 4:**
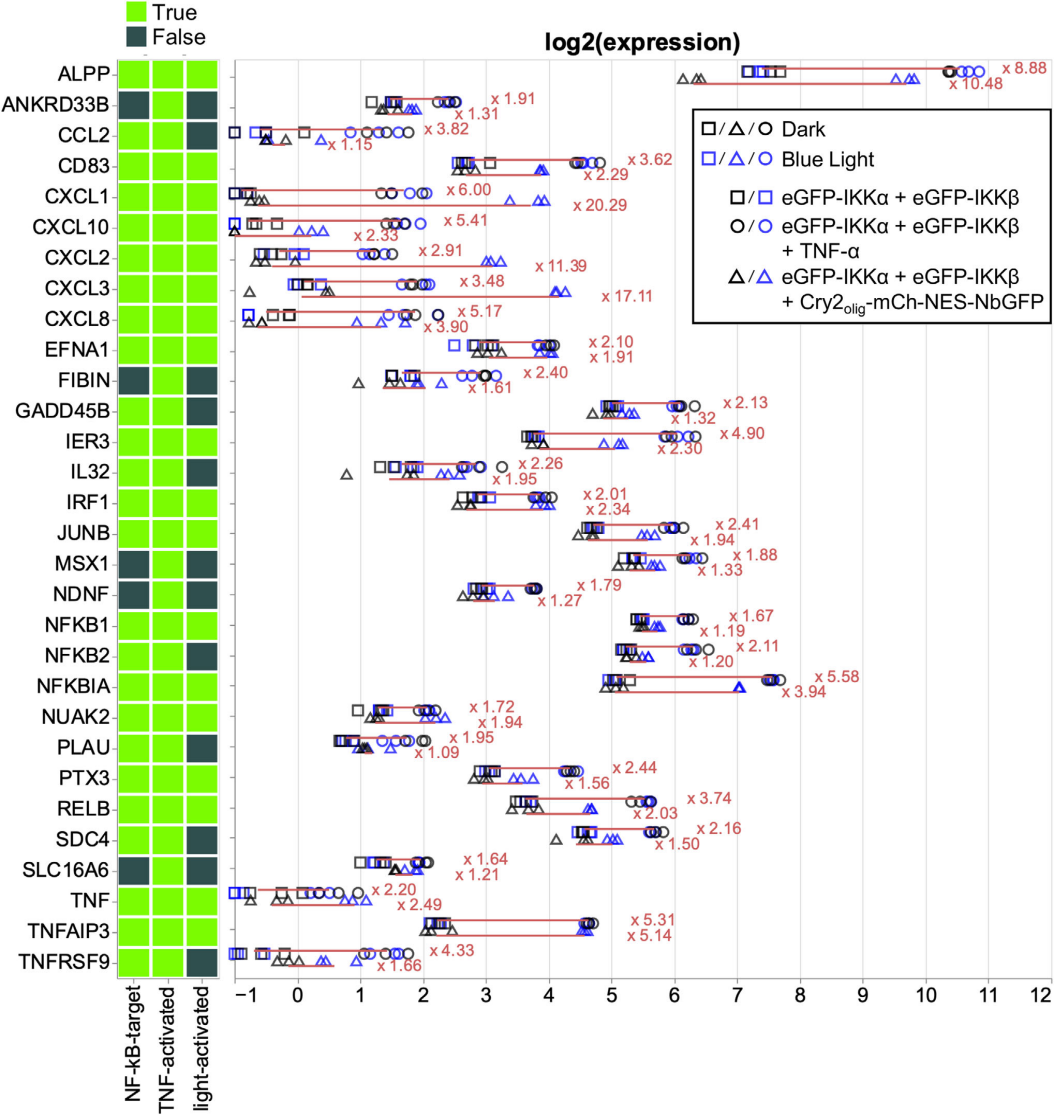
Optogenetic activation of endogenous NF‐κB target genes. HEK‐293T cells were transfected with the indicated constructs. Twenty‐four hours after transfection, indicated samples were stimulated with 20 ng mL^−1^ TNF‐α. Simultaneously, blue‐light illumination (5 µmol m^−2 ^s^−1^) was started. Three hours later, cells were lysed, and total RNA was extracted and subjected to RNAseq (*N* = 3 replicates per sample). Genes with a fold activation > 1.61 and a Benjamini–Hochberg‐corrected *p*‐value (false discovery rate) < 0.05 between the means of ±TNF‐α or D/BL conditions were considered significantly upregulated. Genes marked with false (dark green) in the NF‐κB target category are secondary targets. Log2 expression for each gene and replicate is shown. Red lines indicate the fold induction between the means, numbers are the fold inductions.

After establishing and characterizing our optogenetic toolbox for NF‐κB‐specific reporter gene expression, we investigated if our system could also activate endogenous NF‐κB target genes. We used Cry2_olig_‐mCh‐NES‐NbGFP for eGFP‐IKKα and β clustering and compared the capability of this configuration to activate NF‐kB target genes in comparison to TNF‐α‐mediated stimulation in HEK‐293T cells. We extracted total RNA after 3 h of TNF‐α stimulation or 3 h of blue‐light or dark treatment and analyzed gene expression in the samples via RNA sequencing. We found 30 genes significantly upregulated by TNF‐α stimulation and 18 genes significantly upregulated by blue‐light illumination. All genes were previously reported to be targets of NF‐κB signaling or secondary targets,^[^ ^46^, ^47^, ^48^, ^49^ ^]^ indicating that the optogenetic approach is indeed capable of specifically activating NF‐κB target genes. Notably, not all TNF‐α‐induced genes were also activated by blue light. However, we observed that these genes were not strongly activated by TNF‐α either or secondary targets. The fold induction of many genes was slightly lower for blue light than for TNF‐α; therefore, blue light‐mediated activation might not have been strong enough in these cases or, for the example of the secondary targets, not long enough to be activated over an intermediate step. Alternatively, this might be the result of different activation kinetics that could result in the maximum expression of different genes at different time points (**Figure** **4**). We furthermore verified the RNAseq‐based results by analyzing three NF‐κB target genes (*NFKBIA, TNFAIP3*, and *CXCL1*) via RT‐qPCR. Cry2_olig_‐mCh‐NES‐NbGFP‐induced clustering of eGFP‐IKKα and eGFP‐IKKβ activated all three target genes to a comparable or even higher extent than TNF‐α stimulation (Figure S4, Supporting Information).

In conclusion, the here‐presented system is capable of potently, specifically, and reversibly activating NF‐κB signaling by blue‐light induced clustering of IKKα and β, independent from upstream receptor stimulation.

## 3 Conclusion

The spatial and temporal organization of the cell is of fundamental importance for all biological processes, including the regulation of signaling pathways. The importance of protein oligomerization, scaffolds, or other mechanisms that generate multivalency has come to special interest as many protein functions rely on the precisely regulated formation of higher‐order assemblies and of phase separation.^[^ ^50^, ^51^ ^]^ Furthermore, signal transduction is not a linear process but rather a network of tightly regulated signaling patterns that is integrated into dynamic outputs.^[^ ^52^ ^]^ The gene expression output of the NF‐κB pathway differs depending on the inflammatory stimuli: TNF‐α treatment leads to the expression of inflammatory genes,^[^ ^53^, ^54^ ^]^ whereas LPS stimulation induces the expression of genes required for the adaptive immune response.^[^ ^55^, ^56^ ^]^ Investigation of the differences in signaling patterns that lead to this differential output requires the development of sophisticated, well‐controllable, and fine‐tunable research tools that can, for example, be provided by optogenetics.^[^ ^57^, ^58^ ^]^ In comparison to other pathways such as the MAPK pathway, NF‐κB signaling has only recently moved into the focus of optogenetic control. One approach is to control the NF‐κB transcription factors directly. This was done by Osimiri et al., who used a combination of the LOVTRAP and an AsLOV‐based nuclear import tool to shuttle RelA between the cytoplasm and nucleus, mimicking the oscillating dynamics of the transcription factor and determining the resulting downstream gene activation patterns.^[^ ^59^ ^]^ Other groups targeted MyD88 and TRAF6, which act downstream of toll‐like receptor or interleukin‐1 receptor‐induced signaling, for light‐inducible, Cry2, or Cry2_olig_‐mediated clustering.^[^ ^18^, ^29^ ^]^ Thereby, they could identify IRAK1 as an input dosage sensor for toll‐like receptor and interleukin‐1 receptor‐mediated NF‐κB signaling.^[^ ^29^ ^]^ Further tools activate NF‐κB signaling via Cry2‐mediated clustering of STING (Table S1, Supporting Information).^[^ ^39^, ^60^ ^]^ However, the IKK complex is the central signal integrator of canonical NF‐κB signaling and operates downstream of all major NF‐κB signaling‐inducing receptors.^[^ ^35^ ^]^ Therefore, our tool has the potential to mimic all the different upstream activation mechanisms.

Endogenously, IKKα and β are activated by phosphorylation in their T‐loops. For this process, two mechanisms and their relative importance are still being discussed:^[^ ^61^ ^]^ First, phosphorylation by the upstream kinase TAK1^[^ ^62^, ^63^ ^]^ and second, auto‐transphosphorylation between IKK α and β upon close spatial interaction.^[^ ^64^, ^65^ ^]^ Recently, it has been shown that both the IKK complex and TAK1 are recruited into NEMO/polyUb biomolecular condensates, which supports, according to Du et al., IKK phosphorylation by TAK1.^[^ ^31^ ^]^ However, Bagnéris et al. recently showed, how the viral oncoprotein vFLIP is sufficient to activate the IKK complex by stabilizing a multimeric configuration that brings the IKKs in close proximity and enables activation by trans‐autophosphorylation.^[^ ^66^ ^]^ Furthermore, it has been shown that enforced homooligomerization of IKKα or β via three repeats of the chemical dimerizer FPK is capable of activating NF‐κB, as well.^[^ ^43^ ^]^ This is in line with the observations from the optogenetics‐based clustering as performed in this study (Figure S3, Supporting Information). Cry2‐induced clustering could facilitate a similar mechanism, but full activation might not be achieved due to the lack of TAK1‐induced phosphorylation or other unknown mechanisms (Figure 2B; Figure S2, Supporting Information). We showed that optogenetic clustering was capable of activating downstream NF‐κB signaling by detecting phosphorylation of eGFP‐IKKα, eGFP‐IKKβ, and endogenous IκBα as well as IκBα degradation after blue‐light illumination (Figure 2C).^[^ ^20^ ^]^

Due to the role of phase separation in IKK complex assembly^[^ ^31^, ^32^ ^]^ we hypothesized that inducing phase separation synthetically might benefit the NF‐κB pathway activation. However, the FUS_N_‐containing constructs that should phase separate after blue‐light illumination^[^ ^67^ ^]^ did not outperform Cry2_olig_‐mCh‐NES‐NbGFP, which was also capable of inducing large assemblies (Figure 1).^[^ ^70^, ^71^, ^72^ ^]^ The approach described in this study enables testing of the effect of oligomerization‐induced IKK/NF‐kB signaling without the need for chemical stimulation in a rapidly inducible, non‐invasive, and reversible manner (Figures 2, 3). Finally, our RNAseq analysis showed that the system is capable of specifically activating a set of endogenous NF‐κB target genes comparably to TNFα stimulation (Figure 4).

In conclusion, the here‐developed optogenetic approach enables the systematic testing for the optimal homo‐ or heteromeric clustering state of a target protein. It should be considered that the optimum may vary significantly depending on the target's intrinsic properties, its function in the pathway, or the desired goal of the user. Harnessing the advantages of light as an easily tunable stimulus, these clustering constructs could be used in the future to test different signaling input strengths, lengths, or frequencies, representing a sophisticated approach to decipher the complex signaling dynamics of NF‐κB signaling or other important pathways.

## 4 Experimental Section

### Cloning

All plasmids generated in this study were cloned with Gibson Assembly^[^ ^68^ ^]^ or Aqua Cloning.^[^ ^69^ ^]^ Detailed plasmid descriptions are listed in Table S2 (Supporting Information). Linkers were inserted with oligonucleotides and PCR. For constructs that contain multiple repeats, such as response elements in promoters, *E. coli* was grown at 30 °C. All plasmids were verified with Sanger sequencing.

### Cell Culture and Transfection

HEK‐293T cells (DSMZ, catalog no. ACC 635) were cultivated at 37 °C and 5% CO_2_ in Dulbecco's modified Eagle's medium (DMEM, PAN Biotech, catalog no. P04‐03550) supplemented with 10% (v/v) fetal calf serum (FCS, PAN Biotech, catalog no. P30‐3602) and 1% (v/v) penicillin‐streptomycin solution (PAN Biotech, catalog no. P06‐07100). Cells were passaged when reaching a confluency of ≈80% every 2–3 days. All experiments were conducted in multi‐well plates as described below. Cells were transfected 24 h after seeding with polyethyleneimine (PEI, 1 mg mL^−1^ in H_2_O, pH 7, Polyscience, catalog no. 23966‐1). To do so, the total volume of Opti‐MEM (Thermo Fisher Scientific, catalog no. 22600‐134) was split into two mixes: DNA‐mix and PEI‐mix (exact amounts are described below for each experiment scale), which were prepared separately. Afterward, the two solutions were combined, vortexed immediately for 15 s, and then incubated for 15 min at room temperature. DNA/PEI mixes were then applied to the cells dropwise. For optogenetic experiments, cells were then kept in darkness. Plasmid amounts and combinations for each experiment are described in Table S3 (Supporting Information).

### Reporter Assays

Firefly luciferase assays were performed in black, clear‐bottom 96‐well plates (µCLEAR, Greiner Bio‐One, catalog no. 655090). 15000 cells per well in 100 microliters of DMEM were seeded 24 h prior to transfection. Transfection mixes contained 125 ng DNA and 0.41 µl PEI in 20 µl Opti‐MEM per well. Illumination (5 µmol m^−2^ s^−1^) of optogenetic experiments was started 8 h after transfection using the optoPlate‐96^[^ ^70^ ^]^ equipped with 470 nm LEDs (Würth Elektronik, MPN: 150141RB73100). The optoPlate‐96 was programmed using the optoConfig‐96.^[^ ^71^ ^]^ Stimulation of the controls was performed 8 h after transfection with 20 ng mL^−1^ TNF‐α (Merck, cat. no.: H8916). Cells were lysed 24 h later by addition of 100 µL lysis buffer (25 mm Tris/HCl, pH 7.8, 1% Triton X‐100, 15 mm MgSO_4_, 4 mm ethylene glycol tetraacetic acid (EGTA), 1 mm DTT) per well and 5 min incubation at room temperature (RT). Cells were resuspended by pipetting up and down carefully, and 40 µm lysate of each well was transferred into a white flat‐bottom 96‐well plate (Corning Incorporated, catalog no. CORN3912). Afterward, the plate was centrifuged for 30 s at 1200 rpm. To measure firefly luciferase activity, 20 µL firefly substrate (20 mm Tricine, 2.67 mm MgSO_4_, 0.1 mm EDTA, 33.3 mm DTT, 0.52 mm ATP, 0.27 mm Acetyl‐CoA, 5 mm NaOH, 0.264 mm MgCO_3_, 0.47 mm luciferin) per well was added. The measurement was started immediately using either a Synergy 4 multimode microplate reader (BioTek Instruments Inc.) or a SpectraMax iD5 microplate reader (Molecular Devices GmbH). Program: 10 s shaking, 1000 ms integration time, endpoint measurement. For the calculation of fold induction or fold signal amplification, only samples that were grown and measured on the same plate were compared.

### Flow Cytometry

HEK‐293T cells were seeded and transfected as described above. After 32 h in darkness, the media was removed, cells were detached with 50 µL trypsin/EDTA solution (PAN Biotech, catalog no. P10‐023500), and carefully resuspended in 150 µL PBS supplemented with FCS (2% (v/v)). Afterward, cells were transferred to a transparent U‐bottom 96‐well plate (Greiner, catalog no. 650161) and kept on ice until analysis with an Attune NxT flow (Thermo–Fisher Scientific). eGFP was detected with the BL1 configuration (excitation with a 488 nm laser, detection with a 530/30 nm emission filter) and mCherry with the YL2 configuration (excitation with a 561 nm laser, detection with a 620/15 nm emission filter). Cells were analyzed at a flow rate of 200 µL min^−1^. For analysis with FlowJo‐v10 first live cells (determined via forward (FSC) and side (SSC) scatter), then single cells, and then fluorescent protein (eGFP or mCherry) positive cells were gated. The median fluorescence intensity (MFI) of the fluorescent protein positive population was determined and used for comparison of the expression levels.

### Western Blot

For Western blot experiments, 84 000 HEK‐293T cells were seeded into black, clear‐bottom 24‐well plates (µ‐Plate ibiTreat, ibidi, catalog no. 82426) 24 h prior to transfection. Transfection mixes contained 500 ng DNA and 1.65 µL PEI in 100 µL Opti‐MEM per well. Illumination (5 µmol m^−2^ s^−1^) of cells was started at the indicated durations before lysis using the optoPlate‐96. Twenty‐four hours after transfection, all conditions of the experiment were lysed simultaneously by addition of 75 µL lysis buffer (20 mm Tris‐HCl (pH 7.5), 1 mm EDTA (pH 8), 100 mm NaCl, 0.5% Triton X‐100, 0.1% (w/v) SDS, Halt protease and phosphatase inhibitor cocktail, Thermo Scientific, catalog no. 78443) per well and 5 min incubation on ice. Samples were subjected to a freeze–thaw cycle (−80 °C), transferred into a microcentrifuge tube, and centrifuged for 10 min at 12 000 g and 4 °C. The supernatant was mixed with SDS loading buffer (final concentration: 2.5% (v/v) 2‐mercaptoethanol, 0.01% (w/v) bromophenol blue, 10% (v/v) glycerol, 2% (w/v) SDS, 62.5 mm Tris) and incubated at 95 °C for 5 min. Eight microliters of each sample was subjected to SDS‐PAGE and transferred onto a PVDF membrane. Membranes were incubated in blocking solution (TBS‐T (TBS (50 mm Tris, 150 mm NaCl, pH 7.4) with 0.1% (v/v) Tween‐20) with 5% (w/v) BSA (Carl Roth, catalog no. T844) for 1 h. Primary antibodies anti‐IκBα (Cell signaling, catalog no. 4814), anti‐pIKKα/β (S176/180) (Cell signaling, catalog no. 2697), and anti‐pIκBα(S32/36) (Cell signaling, catalog no. 9246) were diluted 1:1000 in blocking buffer. Anti‐mCherry (Cell signaling, catalog no. 43590) was used at a dilution of 1:2000, and anti‐GAPDH (Cell signaling, catalog no. 5174) and anti‐GFP (Cell signaling, catalog no. 2956) at a dilution of 1:5000 in blocking buffer. Primary antibody incubation was performed at 4 °C overnight. Secondary anti‐rabbit IgG HRP‐linked antibody (Cell signaling, catalog no. 7074) and anti‐mouse IgG HRP‐linked antibody (Cell signaling, catalog no. 7076) were diluted 1:3000 in blocking buffer. Membranes were washed four times with TBS‐T for 10 min each and incubated with secondary antibody for 1 h. Membranes were washed again four times with TBS‐T. ECL solution was added onto the membrane and chemiluminescence was detected in an ImageQuant LAS‐4000 mini system (GE Healthcare, catalog no. 28‐9558‐13).

### Microscopy

For microscopy experiments, cells were grown on collagen‐coated glass coverslips (Carl Roth, catalog no.: YX03.2) in 24‐well format (Corning, catalog no. CORN3524). For coating, 500 µL rat tail collagen I (50 µg mL^−1^ diluted in 25 mm acetic acid, Thermo Fisher Scientific, catalog no. A1048301) was added to each well for one hour at RT. Then, wells were washed three times with 500 µL PBS, and 75 000 cells per well were seeded. Transfection mixes for the 24‐well format contained: 750 ng DNA and 2.4 µL PEI in 100 µL OptiMEM per well. Illumination (5 µmol m^−2^ s^−1^) was started 8 h after transfection for 24 h in ventilated boxes containing microcontroller‐regulated illumination panels with 460 nm LEDs (LED Engin, MPN: LZ1‐10B202‐0000). Controls were kept in identical boxes but in darkness. For fixation, the medium was removed, and 200 µL methanol‐free paraformaldehyde (PFA, Science Services, catalog no. E15714‐S; 4% diluted in PBS v/v) was added under dim, green safe‐light. The samples were incubated for 15 min at RT and in darkness, then PFA was removed, and cells were washed twice with 500 µL of PBS. For the staining of nuclei, 500 µL 0.2 µg mL^−1^ 4′,6′‐diamidino‐2‐phenylindole (DAPI, Merck, catalog no. D9542) in PBS was added for 15 min at RT. Cells were washed twice afterward with 500 µL PBS and mounted on microscopy slides onto 8.5 µL Mowiol mounting medium (2.4 g of Mowiol, 6 g of glycerol, 6 ml of H_2_O, and 12 mL of Tris/HCl (pH 8.5)). Samples were dried and then additionally fixed with transparent nail polish. Images were acquired with a Zeiss LSM 880 laser scanning confocal microscope equipped with a 63x Plan‐Apochromat oil objective (NA 1.4) in z‐stacks with 1 µm distance. DAPI was imaged with the 405 nm laser, eGFP with the 488 nm laser, mCherry with the 561 nm laser, and iRFP670 with the 633 nm laser. All images are displayed as cutouts of the maximum intensity projections with equally adjusted intensities.

### RNAseq and Reverse Transcription Quantitative PCR (RT‐qPCR)

For RNAseq and RT‐qPCR experiments, 90 000 HEK‐293T cells were seeded into 24‐well plates, transfected as described above for the 24‐well format, and kept in darkness for 24 h. Afterward, cells were illuminated (460 nm, 5 µmol m^−2^ s^−1^) in the boxes described above for 3 h, controls were kept in darkness. Simultaneously, TNF‐α (20 ng mL^−1^) was applied to the control group. Afterward, cells were harvested, and RNA was isolated using the RNeasy plus micro kit (Qiagen, cat. no.: 74034), according to the manufacturer's protocol. RNA integrity was verified with an agarose gel, and 260/280 and 230/280 ratios were measured with a nanodrop 1000 (Thermo Fisher Scientific). RNAseq was performed by BGI Tech Solutions (Hong Kong) Co., after which RNA integrity was again verified with a Bioanalyzer (Agilent 2100 Bioanalyzer).

For RT‐qPCR, cDNA was generated using the High‐Capacity Kit (Applied Biosystems, cat. no. 4368814) according to the manufacturer's protocol using 1 µg RNA. cDNA was diluted 1:3.

Primer sequences for the three NF‐κB target genes were chosen from literature: (*NFKBIA*, oAF469: 5′‐ATGTCAATGCTCAGGAGCCC‐3′ and oAF470: 5′‐GACATCAGCCCCACACTTCA‐3′;^[^ ^72^ ^]^ *TNFAIP3*, oAF467: 5′‐CGTCCAGGTTCCAGAACACCATTC‐3′ and oAF468: 5′‐ TGCGCTGGCTCGATCTCAGTTG‐3′;^[^ ^73^ ^]^ *CXCL1*, oAF471: 5′‐AACCGAAGTCATAGCCACAC‐3′ and oAF472: 5′‐GTTGGATTTGTCACTGTTCAGC‐3′^[^ ^74^ ^]^). *GUS* was selected as housekeeping gene for normalization (oMH703/oAF481, 5′‐CGTCCCACCTAGAATCTGCT‐3′ and oMH702/oAF482, 5′‐TTGCTCACAAAGGTCACAGG‐3′). qPCRs were conducted in technical triplicates, in 10 µL approaches using the PowerTrack SYBR Green Mastermix (Applied Biosystems, cat. no. A46110) according to the manufacturer's protocol (fast cycling mode with default dissociation step). A CFX384 Touch Real‐Time PCR Detection System (BioRad) was used for the measurement. Data was analyzed with the ∆∆Ct method and is displayed as fold changes to the negative controls.

### RNAseq Analysis

The externally provided raw but already quality‐controlled and cleaned data in FASTQ format was analyzed using a method based on gene‐specific gapped k‐mer counting. A unique gapped k‐mer is a discontinuous DNA sequence of length k (here, *k* = 23 in a window of length *w* = 33 using the specific mask ######_##_###___#___###_##_######, where # and _ denoted the 23 considered and 10 ignored positions in a window, respectively) that is specific for a gene in the t2t reference genome,^[^ ^75^ ^]^ i.e., occurs nowhere else in the genome than in that gene. The set of such unique gapped k‐mers was first identified using the hackgap k‐mer counter.^[^ ^76^ ^]^ The number of occurrences of each such k‐mer in each FASTQ sample file was then counted, again using hackgap. Per gene and per sample, k‐mer counts were robustly averaged using trimmed means to yield a measure of gene expression. The resulting count matrix was normalized by a scaling factor to account for slightly varying sequencing depth between samples using quantile regression. In detail, all quantiles between the median (0.5‐quantile) and 0.98‐quantile of the sorted expression values of each sample were averaged over all samples to yield an average quantile curve. Then, the optimal scaling factor (offset in logarithmic space) was computed for each sample to regress the samples' quantiles onto the average curve. The resulting normalized expression matrix was subjected to statistical testing and fold change estimation. Fold changes were estimated as ratios between the geometric means of the three replicates in each condition. The compared conditions were ‐/+ TNF‐α on the one hand and darkness versus blue light (D/BL) on the other hand.

Statistical tests were performed with the scipy.stats^[^ ^77^ ^]^ package (v1.12) of Python (v3.11.7), using a two‐sample *t*‐test with unequal variance, combined with Benjamini–Hochberg *p*‐value multiple testing correction for false discovery rate (FDR) control (functions ttest_ind, false_discovery_control). The whole analysis workflow was implemented with Snakemake.^[^ ^78^, ^79^ ^]^

Genes with a fold change > 1.61 and a false discovery rate < 0.05 between the means of −/+ TNF‐α or D/BL conditions were considered significantly upregulated. NF‐κB targets and secondary targets were determined according to the list of NF‐κB target genes provided by the Gilmore Laboratory (Boston University),^[^ ^46^ ^]^ the GeneCards database,^[47]^ and the Enrichr gene list enrichment analysis tool.^[^ ^48^, ^49^ ^]^

### Statistical Analysis

All bar plots from luciferase assays or flow cytometry show means ± SD and single data points. The sample size *N* is indicated in each figure legend. The method of statistical analysis is indicated in each figure legend and was performed either with Microsoft Excel 2016 or GraphPad Prism (9.2.0 or 10.4.0). Data was considered statistically significant if *P* ≤ 0.05. *P*‐values were encoded as follows: ^*^ *P* ≤ 0.05; ^**^ *P* ≤ 0.01; ^***^ *P* ≤ 0.001; ^****^ *P* ≤ 0.0001. RNAseq data shows single data points and fold inductions. Detailed processing and analysis are described above in the Experimental Section. Analyses were performed with Microsoft Excel 2016. Graphs were generated with GraphPad Prism 9.2.0 or 10.4.0. Images were analyzed with ImageJ 2.3/1.53q, including the Biovoxxel Toolbox v2.6.0.^[^ ^80^ ^]^ FACs data were analyzed and plotted using FlowJo‐v10. All schemes were created with BioRender.com.

## Conflict of Interest

The authors declare no conflict of interest.

## Author Contributions

A.A.M.F., M.M.K., M.B., and M.M.G. performed experimental work. A.A.M.F., M.M.K., M.B., G.R., S.R., and W.W. analyzed the results. A.A.M.F., S.R., and W.W. wrote the manuscript. S.R. implemented the RNA‐seq analysis method. M.F. and B.G. contributed to the design of the study and data interpretation. W.W. supervised the work.

## Supporting information



Supporting Information

## Data Availability

The data that support the findings of this study are available from the corresponding author upon reasonable request.
