# Supplementary material for: Activation of NF‐κB Signaling by Optogenetic Clustering of IKKα and β
Source: Adv Biol (Weinh). 2025 Jul 29;9(9):e00384. doi: 10.1002/adbi.202400384 (PMC12447120; doi:10.1002/adbi.202400384)
Supplement: Supplementary file 1 — Supporting Information [file ADBI-9-e00384-s001.pdf]

ADVANCED BIOLOGY

Supporting Information

for *Adv. Biology*, DOI 10.1002/adbi.202400384

Activation of NF-κB Signaling by Optogenetic Clustering of IKKα and β

*Alexandra Anna Maria Fischer, Markus Michael Kramer, Miguel Baños, Merlin Moritz Grimm, Manfred Fliegauf, Bodo Grimbacher, Gerald Radziwill, Sven Rahmann and Wilfried Weber**

Supporting Information

Activation of NF-κB signaling by optogenetic clustering of IKKα and β

*Alexandra A.M. Fischer, Markus M. Kramer, Miguel Baños, Merlin M. Grimm, Manfred Fliegauf, Bodo Grimbacher, Gerald Radziwill, Sven Rahmann, Wilfried Weber**

*Corresponding author E-Mail: wilfried.weber@leibniz-inm.de

Table of contents

Supplementary Figure 1:	Expression levels of the four clustering modules	3
Supplementary Figure 2:	Activation of NF-κB signaling by TNF-α stimulation	4
Supplementary Figure 3:	Pathway activation by clustering of either eGFP-IKKα or eGFP-IKKβ	5
Supplementary Figure 4:	RT-qPCR analysis of optogenetic activation of endogenous NF-κB target genes	6
Table S1:	Comparison of optogenetic NF-κB activators	7
Table S2:	Plasmids used in this study	8
Table S3:	Transfection conditions of each experiment	9

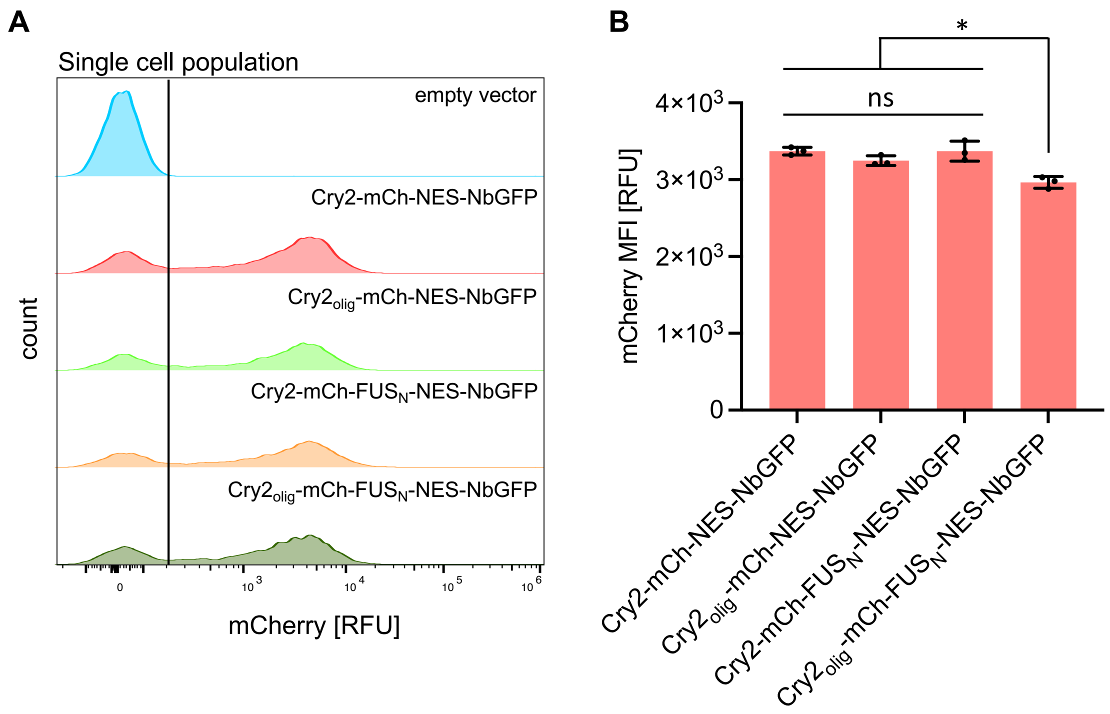

Supplementary Figure 1: Expression levels of the four clustering modules
(**A**) HEK-293T cells were transfected either only with empty vector or with the indicated constructs, eGFP-IKKα, eGFP-IKKβ and an NF-κB-responsive firefly luciferase reporter. Cells were kept in darkness for 32 h and then analyzed by flow cytometry to determine mCherry expression levels. One representative single cell population per condition is plotted (N = 3). (**B**) From the single cell populations in A, mCherry positive cells were gated, the Median Fluorescence Intensity (MFI) determined, and plotted as Mean ± SD (N = 3). Expression levels were compared using an ordinary one-way ANOVA with multiple comparisons. ns = $P \geq 0.05$ $^{*}P \leq 0.05$.

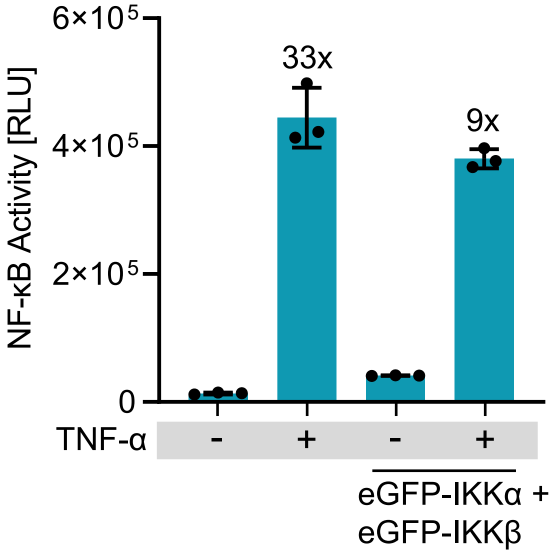

Supplementary Figure 2: Activation of NF-κB signaling by TNF-α stimulation
HEK-293T cells were transfected with an NF-κB-responsive firefly luciferase reporter and either with an empty vector or eGFP-IKKα and eGFP-IKKβ. 8 h after transfection, indicated samples were stimulated with 20 ng/ml TNF-α. 24 h later, firefly luciferase activity was measured. Mean ± SD and single values are shown (N = 3), and fold NF-κB activation between ± TNF-α is shown above the bar.

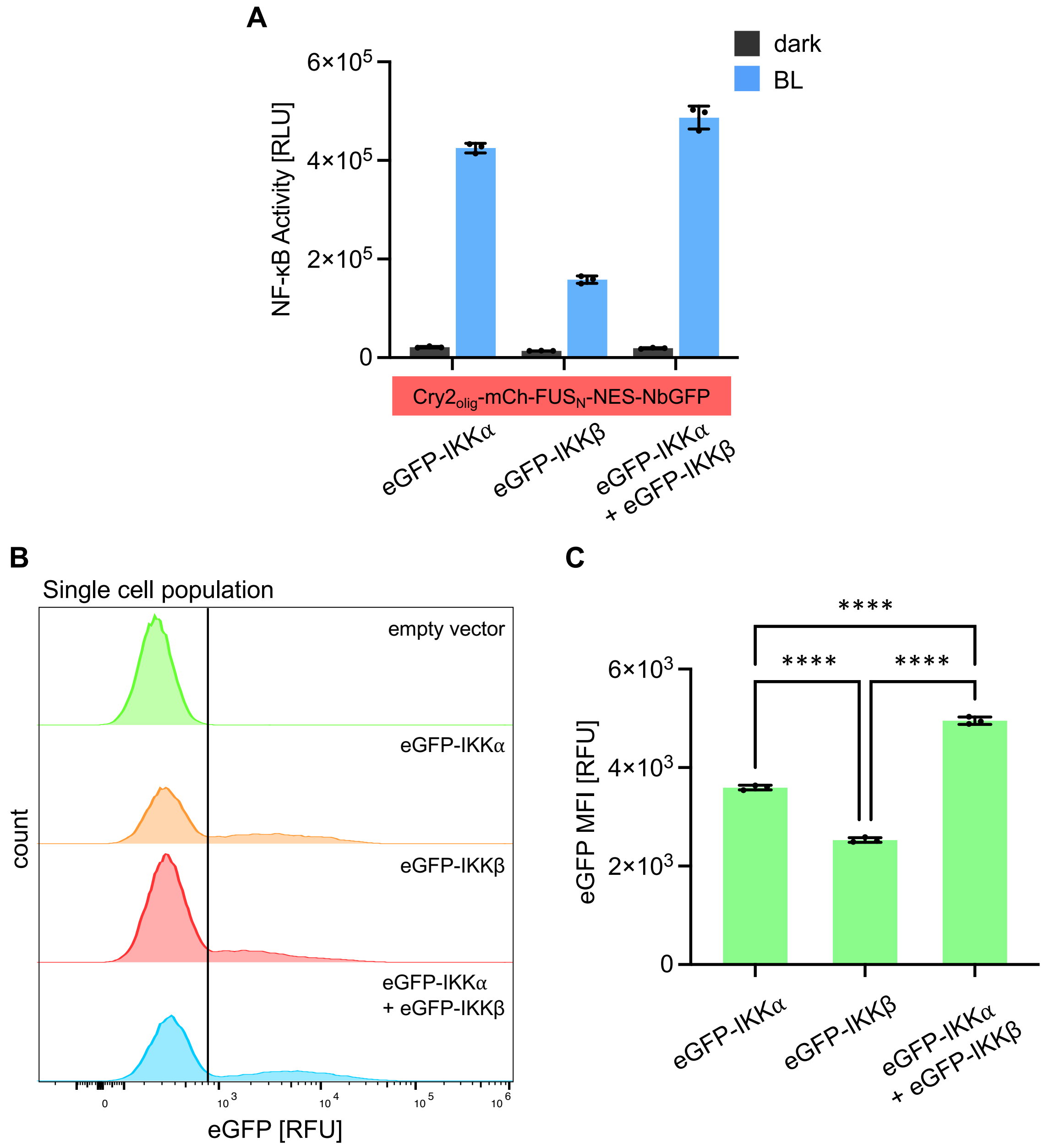

Supplementary Figure 3: Pathway activation by clustering of either eGFP-IKKα or eGFP-IKKβ

(**A**) HEK-293T cells were transfected with the indicated constructs (1 ng plasmid DNA each) and an NF-κB-responsive firefly luciferase reporter. Blue-light illumination (5 µmol m^{-2} s^{-1}) was started 8 h after transfection, and firefly luciferase activity was determined 24 h later. Mean ± SD and fold NF-κB activation is shown (N = 3). (**B**) HEK-293T cells were transfected either with empty vector only or with the indicated constructs, $Cry2_{olig}$-mCh-FUS_N-NbGFP and an NF-κB-responsive firefly luciferase reporter. Cells were kept in darkness for 32 h and then analyzed by flow cytometry to determine eGFP expression levels. One representative single cell population per condition is plotted (N = 3). (**C**) From the single cell populations in B, eGFP positive cells were gated, MFI determined, and plotted here as Mean ± SD (N = 3). Expression levels were compared using an ordinary one-way ANOVA with multiple comparisons $^{****}P \leq 0.0001$.

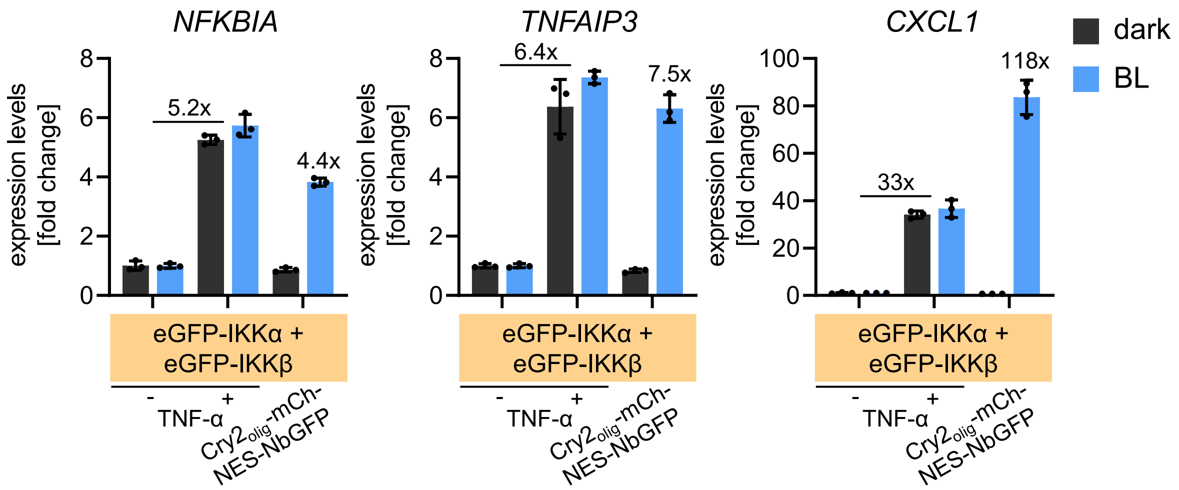

Supplementary Figure 4: RT-qPCR analysis of optogenetic activation of endogenous NF-κB target genes

HEK-293T cells were transfected with the indicated constructs. 24 h after transfection, indicated samples were stimulated with 20 ng/ml TNF-α. Simultaneously, blue-light illumination (5 µmol m^{-2} s^{-1}) was started. 3 h later, cells were lysed, and total RNA was extracted and reverse transcribed. The expression of three NF-κB target genes was analyzed by qPCR. Data was normalized to the housekeeping gene GUS. Mean $2^{\wedge-\Delta\Delta Ct}$ values ± SD are plotted as fold changes to the negative controls (no TNF-α stimulation), N = 3.

Table S1: Comparison of optogenetic NF-κB activity modulators

Tool	Design	Pathway	Reference
Opto-TRAF6	Direct fusion of $Cry2_{olig}$ to TRAF6	Toll-like receptor (TLR) 4 and interleukin receptor 1 (IL-1R) mediated NF-κB signaling	DeFelice et al., 2019
Opto-MyD88	Direct fusion of $Cry2_{olig}$ to MyD88	TLR4 and IL-1R mediated NF-κB signaling	DeFelice et al., 2019
Photo-SMOC	Direct fusion of Cry2 to MyD88 and direct fusion of Cry2 to MAVS combined with shuttling of constitutively active IRF3 in and out of the nucleus via LOVTRAP	TLR and RIG-I-like receptor (RLR) signaling	Tan et al., 2021
LiSmore	Direct fusion of Cry2 or $Cry2_{clust}$ to STING	cGAS-STING mediated NF-κB activation	Dou et al., 2023
Opto-STING	Cry2 fused to a GFP binding nanobody to cluster STING	cGAS-STING signaling activation	Kang et al., 2024
RelA-CLASP	Shuttling of RelA between plasma membrane and nucleus using CLASP (Chen et al., 2020)	Activation of RelA-activated NF-κB target genes	Osimiri et al., 2022
Droplet-RelA	$Cry2_{olig}$-mediated formation of stiff biomolecular condensates around RelA	Inhibition of RelA-activated NF-κB target genes	Fischer et al., 2024
Opto-IKK	Cry2, $Cry2_{olig}$, Cry2-FUS_N or $Cry2_{olig}$-FUS_N fused to a GFP binding nanobody to cluster IKKα and IKKβ	Fine-tunable activation of canoncial NF-κB signaling	This study

Table S2: Plasmids used in this study

Category	Name	Description	Backbone
NbGFP-clustering constructs	pAF352	P_{CMV}-Cry2-mCh-NES-NbGFP-pA	pEGFP-C3
	pAF354	P_{CMV}-Cry2-mCh-FUS_N-NES-NbGFP-pA	pEGFP-C3
	pAF347	P_{CMV}-$Cry2_{olig}$-mCh-NES-NbGFP-pA	pEGFP-C3
	pAF300	P_{CMV}-$Cry2_{olig}$-mCh-FUS_N-NES-NbGFP-pA	pEGFP-C3
NF-κB-POIs	pAF180	P_{CMV}-eGFP-IKKα-pA	pEGFP-C3
	pAF181	P_{CMV}-eGFP-IKKβ-pA	pEGFP-C3
Reporters	NF-kB Firefly luciferase	3x NF-κB-RE-P_{min}-Firefly luciferase reporter-pA	Ref. 42
	pAF504	3x NF-κB-RE P_{min}-SEAP reporter-pA	pMF111
	TK-Renilla luciferase	P_{TK}-Renilla luciferase reporter	pRL-TK
	CMV-Renilla luciferase	P_{CMV}-Renilla luciferase reporter	pRL-CMV
Other	pAF057	empty vector	pEGFP-C3

Table S3: Transfection conditions of each experiment

Figure	Format	Condition	Plasmid name and DNA amount
Figure 1	24-well	eGFP-IKKα + eGFP-IKKβ -/+ TNF-α	pAF180 (6 ng) + pAF181 (6 ng) + NF-κB Firefly luciferase reporter (150 ng) + TK-Renilla luciferase (120 ng) + pAF057 (468 ng)
		eGFP-IKKα + eGFP-IKKβ + NbGFP-clustering construct	pAF180 (6 ng) + pAF181 (6 ng) + pAF352/pAF347/pAF354/pAF300/pAF349/pAF342 (240 ng) + NF-κB Firefly luciferase reporter (150 ng) + TK-Renilla luciferase (120 ng) + pAF057 (228 ng)
Figure 2B, S1, S2	96-well	-/+ TNF-α	NF-κB Firefly luciferase reporter (25 ng) + CMV-Renilla luciferase (20 ng) + pAF057 (80 ng)
		eGFP-IKKα + eGFP-IKKβ -/+ TNF-α	pAF180 (1 ng) + pAF181 (1 ng) + NF-κB Firefly luciferase reporter (25 ng) + CMV-Renilla luciferase (20 ng) + pAF057 (78 ng)
		eGFP-IKKα + eGFP-IKKβ + NbGFP-clustering construct	pAF180 (1 ng) + pAF181 (1 ng) + pAF352/pAF347/pAF354/pAF300/pAF349/pAF342 (40 ng) + NF-κB Firefly luciferase reporter (25 ng) + CMV-Renilla luciferase (20 ng) + pAF057 (38 ng)
Figure 2C, 3	24-well	empty vector	pAF057 (500 ng)
		eGFP-IKKα + eGFP-IKKβ + Cry2$_{olig}$-mCh-NES-NbGFP	pAF180 (4 ng) + pAF181 (4 ng) + pAF347 (160 ng) + pAF057 (332 ng)
Figure 4	24-well	eGFP-IKKα + eGFP-IKKβ -/+ TNF-α	pAF180 (6 ng) + pAF181 (6 ng) + pAF504 (150 ng) + pAF057 (588 ng)
		eGFP-IKKα + eGFP-IKKβ + Cry2$_{olig}$-mCh-NES-NbGFP	pAF180 (6 ng) + pAF181 (6 ng) + pAF347 (240 ng) + pAF504 (150 ng) + pAF057 (348 ng)
Figure S3	96-well	eGFP-IKKα + Cry2$_{olig}$-mCh-NES-NbGFP	pAF180 (1 ng) + pAF349 (40 ng) + NF-κB Firefly luciferase reporter (25 ng) + CMV-Renilla luciferase (20 ng) + pAF057 (39 ng)
		eGFP-IKKα + Cry2$_{olig}$-mCh-NES-NbGFP	pAF181 (1 ng) + pAF349 (40 ng) + NF-κB Firefly luciferase reporter (25 ng) + CMV-Renilla luciferase (20 ng) + pAF057 (39 ng)

		eGFP-IKKα + eGFP-IKKβ + Cry2$_{olig}$-mCh-NES-NbGFP	pAF180 (1 ng) + pAF181 (1 ng) + pAF349 (40 ng) + NF-κB Firefly luciferase reporter (25 ng) + CMV-Renilla luciferase (20 ng) + pAF057 (38 ng)

References

Chen SY, Osimiri LC, Chevalier M, Bugaj LJ, Nguyen TH, Greenstein RA, Ng AH, Stewart-Ornstein J, Neves LT, El-Samad H. 2020. Optogenetic Control Reveals Differential Promoter Interpretation of Transcription Factor Nuclear Translocation Dynamics. *Cell Syst* **11**:336-353.e24. doi:10.1016/j.cels.2020.08.009

DeFelice MM, Clark HR, Hughey JJ, Maayan I, Kudo T, Gutschow M V., Covert MW, Regot S. 2019. NF-κB signaling dynamics is controlled by a dose-sensing autoregulatory loop. *Sci Signal* **12**:eaau3568. doi:10.1126/scisignal.aau3568

Dou Y, Chen R, Liu S, Lee YT, Jing J, Liu X, Ke Y, Wang R, Zhou Y, Huang Y. 2023. Optogenetic engineering of STING signaling allows remote immunomodulation to enhance cancer immunotherapy. *Nat Commun* **14**:1–17. doi:10.1038/s41467-023-41164-2

Fischer AAM, Robertson HB, Kong D, Grimm MM, Grether J, Groth J, Baltes C, Fliegauf M, Lautenschläger F, Grimbacher B, Ye H, Helms V, Weber W. 2024. Engineering Material Properties of Transcription Factor Condensates to Control Gene Expression in Mammalian Cells and Mice. *Small*. doi:10.1002/smll.202311834

Kang DY, Jang Y, Lee H, Lee J, Kang M, Kim DW, Lee Sangkyu, Lee Sanghee. 2024. Optogenetic STING clustering system through nanobody-fused photoreceptor for innate immune regulation. *Sens Actuators B Chem* **399**. doi:10.1016/j.snb.2023.134822

Osimiri LC, Bonny AR, Takagishi SR, Luecke S, Riehs N, Hoffmann A, El-Samad H. 2022. Optogenetic control of RelA reveals effect of transcription factor dynamics on downstream gene expression. *bioRxiv*.

Tan P, He L, Zhou Y. 2021. Engineering Supramolecular Organizing Centers for Optogenetic Control of Innate Immune Responses. *Adv Biol* **5**. doi:10.1002/adbi.202000147
